# Effects of Sadness and Fear on Moral Judgments in Public Emergency Events

**DOI:** 10.3390/bs14060468

**Published:** 2024-05-31

**Authors:** Mufan Zheng, Shiyao Qin, Junhua Zhao

**Affiliations:** Department of Psychology, Wuhan University, Wuhan 430072, China

**Keywords:** sadness, fear, moral judgments, CNI model, public emergency, deontology, utilitarianism

## Abstract

With the rapid development of society and the deteriorating natural environment, there has been an increase in public emergencies. This study aimed to explore how sadness and fear in the context of public emergencies influence moral judgments. This research first induced feelings of sadness and fear by using videos about public emergencies and music, and then used moral scenarios from the CNI model (C parameter: sensitivity to consequences; N parameter: sensitivity to norms; I parameter: general preference for inaction) to assess participants’ moral thinking. In Study 1, participants were divided into a sadness group and a neutral group, while in Study 2, participants were divided into a fear group and a neutral group. During the experiment, participants were exposed to different videos related to public emergencies to induce the corresponding emotions, and emotional music was continuously played throughout the entire experiment. Participants were then asked to answer questions requiring moral judgments. The results showed that based on the CNI model, sadness induced in the context of public emergencies significantly increased the C parameter, without affecting the N or I parameters. Fear increased the I parameter, without affecting the C or I parameters. That is, sadness and fear induced in the context of a public emergency can influence moral judgments. Specifically, sadness increases individuals’ sensitivity to consequences and fear increases the general preference for inaction in moral judgments.

## 1. Introduction

In 2014, two American nurses were accidentally infected with the Ebola virus while aiding in Africa [1,2]. They needed assistance to return to the United States to receive appropriate treatment. However, the Ebola virus is extremely dangerous, and the nurses’ return could potentially expose the entire country to the risk of Ebola infection. Should the two doctors be allowed to return to their own country to save their lives? This is an ethical issue in a public emergency event.

In the past several years, the COVID-19 pandemic has caused significant damage to the whole world. In fact, with the rapid growth of the economy and society, as well as the accelerated changes in the natural environment, public emergencies have frequently occurred worldwide in recent years. The term ’public emergency event’ refers to a sudden occurrence that poses a significant threat and harm to national security, the legal system, social security, public order, and the safety of citizens’ lives and properties in the whole country or certain regions, causing massive casualties, property losses, and social impacts [3]. Radical changes caused by the COVID-19 pandemic affect what is considered right or wrong underlying moral judgments [4]. The decisions made by various parties involved in public emergencies, especially those related to ethical and moral considerations, greatly influence the development and outcome of the events. During a public emergency, individuals often undergo strong negative emotions. Studies have revealed that fear and sadness were commonly experienced by people during the COVID-19 pandemic [5,6]. Sayegh et al. [7] posit that the emotions of decision-makers during crisis situations can significantly impact their perception and interpretation of events, consequently influencing their decision-making process. Additionally, a body of research has demonstrated the influence of emotions on moral judgments [8,9,10]. Therefore, this study aimed to explore how negative emotions induced in the context of public emergency events influence moral judgments.

### 1.1. Moral Judgments

The most widely used paradigm in the moral judgment research area is the moral dilemma, with the most classic example being the “trolley dilemma” [11]. The traditional moral dilemma paradigm typically presents a scenario where participants must decide whether to take action that sacrifices a few individuals to save a larger number of people. Based on participants’ final choices, their utilitarian or deontological moral principles can be inferred [12]. Utilitarianism highlights the consequences of moral choices for overall well-being, while deontology is mainly concerned with whether ethical choices are consistent with moral norms and rules [13]. Despite the widespread use of the traditional moral dilemma paradigm, it has also faced criticism. One major critique is that the traditional paradigm fails to accurately quantify inclinations towards utilitarianism and deontology. This paradigm treats utilitarianism and deontology as opposing principles, assuming that if one is enhanced, the other is weakened. As a result, the observed differences in outcomes are ambiguous, and the results may reflect only the choices influenced by utilitarian inclinations, deontological inclinations, or a combination of both [14].

In response to the limitations of the traditional paradigm, Conway and Gawronski proposed a new research paradigm called the process dissociation paradigm [15]. The aim of the process dissociation paradigm is to manipulate the costs and benefits of different consequences resulting from action choices to independently quantify utilitarian and deontological inclinations. This research paradigm no longer views utilitarianism and deontology as mutually exclusive, but as coexisting and operating in parallel. However, this paradigm also has certain limitations. For example, it only considers situations where norms prohibit certain actions and does not consider moral judgments in situations where norms endorse certain actions [16].

### 1.2. CNI Model

To overcome the apparent limitations of previous research paradigms, Gawronski et al. [13] proposed the CNI model, which is based on a data modeling approach commonly used in empirical ethics and social psychology called the multinomial processing tree model [17]. The CNI model reframes utilitarianism and deontology, representing them by using three parameters: (1) sensitivity to consequences (C parameter), (2) sensitivity to norms (N parameter), and (3) general preference for inaction versus action irrespective of consequences and norms (I parameter). Higher estimated values of C and N indicate greater sensitivity to consequences and norms, respectively. A higher estimated value of I suggests a lower general inclination towards action, indicating a greater preference for inaction.

The CNI model has been increasingly adopted by researchers and has received substantial empirical support in the field of moral judgments. Researchers have used the CNI model to investigate the influence of various individual physiological characteristics, such as gender [13], testosterone [18], chronic stress [19], incidental emotions [20], personality traits [21], and so on. Therefore, this study also adopted CNI model to study how sadness and fear induced by public emergency events affect moral judgments.

### 1.3. Emotion and Moral Judgments in Public Emergency Events

Some researchers have also investigated moral judgments in public emergencies, especially during the COVID-19 pandemic. For example. Mazza et al. discussed the relationship between the level of perceived stress and the moral judgments of university students and workers in Italy [22]. Another study conducted by Francis and McNabb compared utilitarian choices before and during the COVID-19 pandemic [4]. According to previous research in the field of ethics, it has been found that emotional factors play a very important role in moral judgments [9]. However, there is little research on the impact of emotions on moral judgments in the context of public emergency events.

Sadness and fear are two important types of emotions in moral judgments and public emergency events. In public emergency events, individuals typically experience five stages: shock (fear), heroism (altruism), sadness (internalization), anger (externalization), and rebuilding normalcy [23]. Researchers have found that people experience sadness and fear frequently and intensely during public emergency events [5,6].In the moral dilemma of “harming to save”, participants mainly experience fear and sadness [9,10].

Previous research has shown that although sadness and fear are both negative emotions, they have significantly different effects on individuals’ behavior and cognition. Fear primarily leads to avoidance tendencies [24], while the impact of sadness is more complex and may influence individuals’ cognitive styles, behavioral tendencies, and emphasis on different moral theories [25,26]. Therefore, this study selected sadness and fear as the independent variables in the research. Studying these two different negative emotions separately can help us to better understand individuals’ moral judgments in public emergency events.

Researchers have found that incidental emotions tend to carry over from one situation to another and influence other decisions [27]. According to Greene’s dual-process model of moral decision-making, general negative emotional experiences lead to more deontological moral choices. When individuals perceive rich details of harm to victims in moral contexts [28], they tend to make more deontological judgments. Negative emotions are significantly associated with deontological choices [9], while positive emotions increase utilitarian moral judgments [29]. Gawronski once studied how incidental happiness, sadness, and anger influence responses to moral dilemmas based on the CNI model [20]. They found that incidental happiness reduced sensitivity to moral norms. Nevertheless, incidental sadness and incidental anger did not influence any factors in moral dilemma judgments.

Specifically regarding fear, it increases individuals’ uncertainty and leads to a tendency to avoid actions in decision-making. Fear causes people to exhibit risk aversion in behavioral decisions [30]. Schubert proposed that fear is always accompanied by avoidance tendencies [31]. Thus, we expected that individuals induced with fear in public emergency events will exhibit a preference for inaction in moral decision-making.

For sadness, the situation is more complex. Firstly, like fear, sadness is believed to increase individuals’ avoidant tendencies in behavior [25,30]. Secondly, some studies suggest that sadness enhances individuals’ cognitive processing abilities. Alloy and Abramson found that sadness makes people wiser, more thoughtful, and cautious [32]. Sadness leads to more systematic processing [33], leading individuals to rely on complex concepts for judgment and reasoning. Thirdly, sadness has been found to be associated with increased attention to outcomes. Wright and Bower found that individuals in a sad emotional state significantly overestimate negative events and underestimate positive events [34]. Sadness has been found to make individuals more myopic and focused on immediate gains [35]. In summary, we expected that individuals increase their focus on consequences and preferences for inaction when they experience sadness in public emergency events.

### 1.4. Manipulation of Emotion

Although Gawronski’s study found that incidental sadness did not have a significant impact on the C, N, or I parameters, we decided to study the effect of sadness on moral judgments again in the present study [20]. Gawronski mentioned that the reason why sadness did not have an impact on moral choices may be due to the insufficiently strong emotional induction effect of music [20], so we will induce stronger emotion in the present study. Also, we think that the intensity of emotions triggered by public emergencies might be higher than that of ordinary incidental emotions.

Following Gawronski’s study [20], we also chose to have participants listen to music to induce sadness and fear. Koelsch suggests that music can induce unpleasant emotions and has advantages over static images [36]. Music tends to have a stronger effect and induce emotions that last longer compared to those induced by other materials. Furthermore, the emotional experiences elicited by music demonstrate good cross-cultural consistency [37].

However, in order to achieve better induction effects and induce emotions related to public emergency events, the present study combined watching videos and listening to music to arouse emotions. Eldar et al. found that compared to watching a movie or listening to music separately, having participants simultaneously watch a movie and listen to positive (happy) or negative (fearful) music can better activate the participants’ amygdala and ventromedial prefrontal cortex [38]. This means that using multiple emotion induction methods in combination can more effectively induce emotions.

### 1.5. The Present Study

The current study explores how two common types of negative emotions (sadness and fear) during public emergency events influence moral judgments based on the CNI model. Study 1 and Study 2 induced sadness and fear, respectively, using a combination of videos and music, and then assessed participants’ C, N, and I tendencies based on their moral choices in the scenarios from the CNI model of Gawronski’s study. This study hypothesizes that sadness will enhance individuals’ sensitivity to consequence (parameter C) and increase their general preference for inaction (parameter I), while having no significant effect on parameter N. Fear will lead to an avoidance tendency, making individuals less willing to take action, thus increasing their general preference for inaction (parameter I), with no significant effect on the other two parameters.

## 2. Study 1

Using the CNI model, Study 1 aims to explore how sadness influences moral judgments in public emergency events.

### 2.1. Participants

Sample sizes (*n* = 128) were calculated according to the medium effect size (Cohen’s *d* = 0.25) for *t* tests with a desired power level of *p* = 0.80 and a desired alpha error probability of *p* = 0.05. We recruited 132 participants to prevent the occurrence of invalid data. Two participants who had a response time of less than 30 s were excluded, and the final sample included 130 participants with a validity rate of 98.5%. Among them, there were 48 males and 82 females, with a mean age of 21.1 (*SD* = 1.75, age range from 18 to 26). All participants were Chinese, all of whom were undergraduate and graduate students from Wuhan University in China. The participants were recruited through the department’s participant pool. Participants received a reward of CNY 10 (around USD 1.38) for participating in the experiment. All participants had normal hearing and vision. They provided informed consent and voluntarily participated in the experiment. Participants received compensation after the completion of the experiment.

### 2.2. Procedure

Participants were randomly assigned to either the sadness group or the neutral emotion control group. Emotion was the independent variable, and the three parameters of the CNI model for moral decision-making (sensitivity to outcomes, sensitivity to moral norms, and preference for inaction) were the dependent variables.

The experiment took place in a well-lit and quiet lab with computers and headphones. After informing the participants and obtaining their consent, they were instructed to wear headphones and watch a video on a computer to induce emotions. Participants in the sadness induction group watched a news report video about the Wenchuan earthquake titled “A video from 10 years ago, she cried, and I cried too”. Participants in the neutral group watched a news video segment about a number of confirmed cases of COVID-19 in February 2022.

To strengthen the effect of emotion induction, participants then listened to music to arouse emotion, which played continuously throughout the experiment. The music used to arouse sad emotions was the theme song of the famous movie “Schindler’s List”, called “Theme From Schindler’s List”. For the neutral group, the music chosen was “Common Tones in Simple Time” composed by John Adams.

While the music played, participants were required to read moral scenarios from Gawronski’s study [13] and make judgments on 24 of the scenarios. The order of the 24 scenarios was randomized.

Gawronski and colleagues [13] proposed the CNI model of moral judgments, in which they selected six real-life dilemmas covering four combinations of normative (prohibitive or prescriptive) and consequential (greater good or lesser evil) types of situations, resulting in a total of 24 moral dilemma stories. The four categories of moral dilemmas include the following: (a) dilemmas where an action is prohibited by a rule, but the benefits outweigh the costs; (b) dilemmas where an action is prohibited by a rule, but the costs outweigh the benefits; (c) dilemmas where an action is required by a rule, and the benefits outweigh the costs; and (d) dilemmas where an action is required by a rule, but the costs outweigh the benefits [39]. This framework estimates three factors influencing moral dilemma judgments: (a) sensitivity to consequences, (b) sensitivity to moral norms, and (c) a general preference for inaction or action regardless of consequences and norms. While sensitivity to consequences and moral norms align with utilitarianism and deontology, the general preference for inaction is linked to the omission bias, which suggests that harm resulting from action is often viewed as more severe than harm resulting from inaction.

The CNI model assumes that decision-makers follow the principle of serial processing when making moral judgments. When individuals are driven by utilitarian principles, they accept choices in moral dilemmas when the outcome is favorable and reject choices when the outcome is unfavorable. If individuals are driven by deontological principles, they accept options that comply with moral standards and reject proposals that violate those standards. If individuals are not driven by either utilitarian or deontological principles, the decision to accept or reject choices will be influenced by a general tendency towards action or inaction [13].

Subsequently, participants answered two questions about sadness (sad and depressed) to check the effect of emotion induction, with a rating scale ranging from 1 (not at all) to 7 (extremely intense). Also, we used two items to measure fear (scared and fear), two to measure happiness (happy and delighted), and two to measure anger (angry and annoyed). Finally, participants were asked to provide demographic information.

### 2.3. Results and Discussion

The average scores of the sadness emotion were calculated. Consistent with our expectation, participants who underwent sadness manipulation reported experiencing more sadness (*M* = 3.14, *SD* = 1.42) compared to those in the neutral emotional manipulation group (*M* = 2.11, *SD* = 1.27), *t*(128) = 4.35, *p* < 0.001, *d* = 0.763. The emotional manipulation was successful. The participants in the experimental group and the control group did not show differences in their feelings of fear (*M_experiment_* = 2.29, *SD* = 1.13; *M_control_* = 2.02, *SD* = 1.08; *t*(128) = 1.41, *p* = 0.16), anger (*M_experiment_* = 1.88, *SD* = 0.95; *M_control_* = 2.16, *SD* = 0.98; *t*(128) = −1.69, *p* = 0.09), and happiness (*M_experiment_* = 2.05, *SD* = 1.00; *M_control_* = 2.27, *SD* = 0.88; *t*(128) = −1.38, *p* = 0.17). This indicates that the experimental material did not significantly induce other emotions.

The CNI model analysis was conducted using Moshagen’s polynomial modeling software, multiTree [40], and the multiTree template file provided by Gawronski et al. [13] for CNI model analysis. The model calculated the parameters C, N, and I. The CNI model showed a good fit to the data, *G*^2^(2) = 2.338, *p* = 0.311. The purpose of the data was to examine whether the predicted probabilities from the model significantly differed from the empirically observed probabilities in the dataset. In other words, the CNI model only fitted the data when the differences were not statistically significant. The *G^2^* value represents the reported model fit. By substituting *C*1 = *C*2 into the model, Δ*G*^2^(1) = 9.701 (Δ indicates the difference in the model fit being tested), *p* = 0.002, *d* = 0.552, indicating a significant difference between the two groups in the *C* parameter. Specifically, compared to the neutral group, the sadness group showed greater sensitivity to outcomes (see Table 1). By substituting *N*1 *= N*2 into the model, Δ*G*^2^(1) = 0.870, *p* = 0.351, indicating no significant difference between the two groups in the N parameter. This means that there was no significant difference in sensitivity to moral norms between the two groups. By substituting *I*1 *= I*2 into the model, Δ*G*^2^(1) = 0.000, *p* = 0.982, indicating no significant difference between the two groups in the *I* parameter. This means that there was no significant difference in the preference for action/inaction between the two groups.

The study suggests that in the context of public emergency events, sadness can influence moral judgments by increasing individuals’ sensitivity to outcomes. However, sadness do not affect sensitivity to moral norms or preferences for general action/inaction.

## 3. Study 2

In addition to sadness, fear is another common negative emotion experienced during public emergency events. Fear is an acute reaction characterized by a loss of self-control, resulting in non-social and irrational escape behaviors [41]. This behavior is based on the perception of being trapped, a sense of helplessness in the collective, and the resulting personal isolation during crises. Moreover, potential public fear can also trigger “secondary disasters”. Unlike sadness, fear during public emergency events can easily spread through a contagion effect, resulting in collective fear among the population [42]. Therefore, study 2 studied how fear affects moral judgments against the background of public emergency events.

### 3.1. Participants

Sample sizes (*n* = 128) were calculated according to the medium effect size (Cohen’s *d* = 0.25) for *t* tests with a desired power level of *p* = 0.80, and a desired alpha error probability of *p* = 0.05. A total of 128 participants were recruited to participate in this study. Among them, there were 45 males and 83 females, with a mean age of 21.0 (*SD* = 2.50, age range from 16 to 27). All the participants were Chinese and all were undergraduate and graduate students from Wuhan University in China. The participants were recruited through the department’s participant pool. Participants received a reward of CNY 10 (around USD 1.38) for participating in the experiment. All participants had normal hearing and vision. They provided informed consent and voluntarily participated in the experiment. Participants received compensation after the completion of the experiment.

### 3.2. Procedure

Participants were randomly assigned to either the fear group or the neutral emotion group. Emotion was the independent variable, and the three parameters of the CNI model for moral decision-making (sensitivity to outcomes, sensitivity to moral norms, and preference for inaction) were the dependent variables. The experimental procedures in Study 2 were the same as in Study 1.

The materials used for emotion induction were different from Study 1. For the fear induction group, a video of the Wenchuan earthquake captured by surveillance cameras was used, titled “Terrifying Moment–The Wenchuan Earthquake 12 Years Later”. The music used was “Cloudy Day”, produced by Phew. For the neutral emotion group, the same materials as in Study 1 were used for emotion induction. The manipulation check questions were two items relating to fear (fear and scared) with a rating scale ranging from 1 (not at all) to 7 (extremely intense). Also, two items were used to measure sadness (sad and depressed), two to measure happiness (happy and delighted), and two to measure anger (angry and annoyed).

### 3.3. Results and Discussion

The average scores of the fear emotion were calculated. Consistent with our expectation, participants who underwent fear manipulation reported experiencing more fear (*M* = 3.85, *SD* = 1.74) compared to those in the neutral emotion group (*M* = 1.90, *SD* = 1.18), *t*(126) = 7.41, *p* < 0.001, *d* = 1.31. The emotional manipulation was successful. The participants in the experimental group and the control group did not show differences in their feelings of sadness (*M_experiment_* = 2.43, *SD* = 1.15; *M_control_* = 2.09, *SD* = 1.24; *t*(126) = 1.59, *p* = 0.11), anger (*M_experiment_* = 2.32, *SD* = 0.83; *M_control_* = 2.38, *SD* = 0.74; *t*(126) = −0.39, *p* = 0.69), or happiness (*M_experiment_* = 2.04, *SD* = 0.92; *M_control_* = 2.24, *SD* = 0.90; *t*(126) = −1.27, *p* = 0.21). This indicates that the experimental material did not significantly induce other emotions.

CNI model analysis was conducted using the polynomial modeling software multiTree developed by Moshagen [40], along with the multiTree template file provided by Gawronski et al. [13] for CNI model analysis. The parameters C, N, and I were computed. The CNI model showed a good fit to the data, with *G^2^*(2) = 0.983, *p* = 0.612. By substituting *C*1 *= C*2 into the model, Δ*G*^2^(1) = 1.117, *p* = 0.290, indicating no significant difference in the C parameter between the two groups. This suggests that both groups had similar sensitivity to the outcomes. By substituting *N*1 *= N*2 into the model, Δ*G*^2^(1) = 1.164, *p* = 0.281, indicating no significant difference in the N parameter between the two groups. This implies that both groups had similar sensitivity to moral norms. By substituting *I*1 *= I*2 into the model, Δ*G*^2^(1) = 11.861, *p* = 0.008, *d* = 0.587, suggesting a significant difference in the I parameter between the two groups. This indicates that there was a significant difference in the preference for action/inaction between the two groups. Specifically, compared to the neutral group, the fear induction group showed greater inaction inclination (see Table 2). These results suggest that fear influences moral dilemma judgments by increasing the preference for inaction. Fear does not affect sensitivity to moral norms or outcomes.

In previous studies on moral judgments, the general action/inaction tendency was usually not distinguished from utilitarian and deontological tendencies. In the context of sudden public emergency events, the action inclination is an important factor involved in individuals’ moral judgments. It determines the individual’s final moral choices and subsequent actions. By utilizing the CNI model, we were able to explore how fear, a common emotion in public emergency events, influences individuals’ moral judgments. We discovered that fear affects moral judgments through decreasing individuals’ action inclination. This means that when individuals experience fear during public emergency events, they are less inclined to take action. However, fear does not affect sensitivity to moral norms or sensitivity to outcomes.

## 4. General Discussion

This study focuses on how two types of negative emotions, sadness and fear, against the background of sudden public emergency events, influence participants’ moral choices. The results show that participants induced with sadness showed a significant increase in outcome sensitivity compared with those in the neutral emotion group, indicating a greater focus on whether the behavioral consequences yield more benefits than costs. Meanwhile, participants with fear show a significant increase in the general preference for inaction compared with those in the neutral emotion group, indicating a greater reluctance to take action.

We chose the CNI model to discuss how sadness and fear induced by sudden public emergency events affect moral judgments. This model helps us to calculate whether the two types of emotions can affect moral choices and, specifically, through which factors they influence moral judgments. In traditional moral dilemma paradigms, people must choose whether to break moral rules to achieve good outcomes. However, just because someone chooses to sacrifice one life to save others does not mean that they always follow utilitarian principles. It is important to see if people make consistent decisions for different dilemmas to understand their moral beliefs. It is also hard to attribute a decision solely to one type of reasoning, ignoring the other. To address this, Gawronski et al. created the CNI model, which measures how much people care about outcomes (C), moral rules (N), and preferring not to act (I). This model helps people to better understand what factors ultimately govern an individual’s moral choices, and what factor(s) are affected by sadness and fear.

The current research discusses the influence of fear and sadness on moral judgments based on the CNI model and clarifies how these two emotions impact moral thinking processes. This contributes to the understanding of the relationship between negative emotions and moral judgments. In previous studies, the influence of negative emotions on moral judgments has been controversial. According to Greene’s dual-process model of moral judgments [12], general experiences of negative emotions tend to lead to more deontological moral choices [43,44]. However, different specific emotions may have different effects on moral decision-making [43]. In the case of the two emotions being explored in this study, fear tends to lead individuals to exhibit avoidance tendencies in decision-making [31], which may be related to deontological choices in moral dilemmas. However, the situation is more complex for sadness. Researchers believe that sadness increases individuals’ tendencies to inhibition [25,30], which is usually associated with deontological choices in classical moral dilemmas. Nevertheless, some other studies have shown that sadness can lead to stronger cognitive processing abilities [32] and greater attention to outcomes [34]. That means that sadness is also possible to relate to utilitarian moral judgments. Using classical moral dilemma paradigms or process dissociation techniques cannot disentangle the impact of negative emotions, especially sadness, on moral judgments. This study utilizes the CNI model to clarify the role of different factors in moral judgments and effectively address the contradictory findings of previous studies.

Our findings are different from Gawronski et al.’s research [20] into the relationship between three specific incidental emotions (happiness, sadness and anger) and moral judgments based on the CNI model. Their study found that incidental sadness did not have a significant impact on the C, N, or I parameters. There are several possible explanations. In Gawronski et al.’s study, the authors believe that incidental sadness might have had an impact on moral dilemmas, but this effect was too small to be detected in their study because of the sample size. Also, they mentioned that failed emotional induction manipulation may be another reason. They proposed that, compared to the method of using emotional music clips currently used, other methods of dealing with incidental sadness may be more effective in influencing moral dilemma judgments. Therefore, the current study replaced the musical materials used by Gawronski et al. [20] and incorporated video materials to induce sadness, making the induction of sadness more reliable. Meanwhile, the present study focused on sadness and fear induced by public emergency events. The intensity of emotions triggered by public emergencies is higher than that of ordinary incidental emotions. As mentioned earlier, the unique emotional states triggered by sudden public emergency events may lead individuals to make different moral judgments than usual. This is also why we observed the impact of sadness on moral judgments in the present study.

In Study 1, participants induced with sadness showed significantly higher consequence sensitivity (parameter C) than the neutral group. This is consistent with previous research and theories about sadness. Incidental sadness promotes cognitive elaboration by enhancing the motivation for effortful processing [26] or alleviating the demand for cognitive resources [45]. To some extent, the analysis of costs and benefits in utilitarianism is a cognitive effort [46], so incidental sadness may increase consequence sensitivity in a utilitarian way. Some researchers have found a correlation between high emotional intensity and higher consequence sensitivity [21].

Study 2 discussed the impact of fear on moral judgments and found that participants induced with fear show an inaction tendency in moral judgments. Research on emotions and moral judgments mainly focuses on morality-related emotions, such as guilt, disgust, and shame [47,48], and there have been few studies directly investigating the relationship between fear and moral choices. Fear is associated with evaluations of danger or threat, low certainty, and a sense of lack of control over the situation [49]. Schubert proposed that fear is strongly associated with aversion and avoidance tendencies [31]. When participants are induced with fear, they may perceive lower control over the environment and increased uncertainty, making them more likely to choose inhibited choices in moral decision-making. This leads to a significantly higher estimated value of the I parameter compared to the neutral group.

This study focuses on two specific negative emotions, sadness, and fear, which are commonly experienced by individuals in public emergencies, and investigates their impacts on moral judgments. Based on the CNI model in the field of moral decision-making, this study explores the three parameters of moral decision-making tendencies: outcome sensitivity, norm sensitivity, and the general preference for inaction. The study found that sadness and fear, as two negative emotions, have different mechanisms of influence on moral judgments in the context of public emergency events, and their effects are different from the impacts of incidental emotions in daily life.

### 4.1. Practical Implications

Facing public crisis, individuals frequently experience intense negative emotions. Research indicates that feelings of fear and sadness were prevalent among individuals amidst the COVID-19 outbreak [5,6]. According to Sayegh et al. [7], the emotional state of decision-makers in emergency scenarios can greatly affect how they perceive and understand events, ultimately shaping their decision-making processes. Therefore, studying how negative emotions such as fear and sadness affect individual moral judgments during public emergency events can help us understand ethical moral decisions made during public emergency events, and assist people in making better decisions.

### 4.2. Limitations and Future Directions

Although the CNI model helps us thoroughly understand the process of how fear and sadness influence moral judgment, this model still has a limitation [50]. The CNI model lies in its theoretical assumption that decision-makers’ decision-making processes are sequential: decision-makers first consider the outcomes, then they consider the norms without considering the outcomes, and only when they do not consider norms or outcomes do they show a general tendency to accept or reject. However, decision-makers may engage in parallel processing, with three possibilities: considering both the norms and the potential outcomes simultaneously, or first considering whether the decision aligns with the norms and then considering the outcomes, or initially forming a general behavioral attitude and then adjusting it according to the norms or outcome principles [50,51]. Some researchers have proposed methods to address the limitations of the CNI model; for example, the CAN algorithm [52] and pro-CNI algorithm [53].

Gawronski studied how emotions influence moral thinking in a normal state [20], while our research focused on emotions in the context of public emergency events. We aimed to compare our research findings with Gawronski’s original study based on the same theory and method. Therefore, in our study, we adopted the classical calculation method of the CNI model, which facilitates comparison with the previous original study on the same basis. However, future research can use new methods, such as the CAN algorithm, to validate the findings of the current research.

The shortcomings of this study lie in the relatively homogeneous sample of participants, all of whom are university students, without including a more diverse population for research. Public emergency events often have impacts on the entire society. Future research should consider the effects of research findings on a broader population and consider the impact of emotions on moral thinking in different types of people.

The experimental group used news related to the Wenchuan earthquake, while the control group used news related to the COVID-19 pandemic. The participants in the experimental group might be too young to possess significant memories of the crisis event during the 2008 earthquake. However, the control group underwent the recent crisis of the COVID-19 pandemic, resulting in distinct and vivid recollections. This difference in memory retention might impact the comparability of the two sets of materials utilized. Although we believe that human emotions are universal, our research design was more concerned with the emotions contained in and induced by the experimental materials, and we think that the time difference will not affect the participants’ emotional experiences. However, for the sake of rigor and to control for irrelevant variables, future research should try to select materials from the same social events but with different emotional responses and try to eliminate irrelevant factors as much as possible.

In the context of public emergency events, individuals may experience other types of negative emotions in addition to sadness and fear, such as tension when dealing with threats and anxiety about the unknown future, and they may also experience some positive emotions, such as gratitude due to others’ help, admiration for heroes, and so on. Therefore, future research could further discuss the effects of other types of emotions on moral judgments.

## 5. Conclusions

The main objective of this study was to investigate how the emotions of sadness and fear influence moral judgments in the context of public emergency events. The results revealed that sadness increased sensitivity to consequences in moral judgments, while not affecting sensitivity to moral norms and the general preference for inaction. Fear increases the general preference for inaction in moral judgments, while not affecting sensitivity to consequences and moral norms.

## Figures and Tables

**Table 1:** CNI parameter and 95% confidence intervals of the sadness and neutral groups.

Group	C			N			I		
	*M*	*SD*	95%CI	*M*	SD	95%CI	*M*	*SD*	95%CI
Sadness	0.42	0.02	[0.37, 0.46]	0.14	0.04	[0.06, 0.21]	0.48	0.02	[0.45, 0.52]
Neutral	0.31	0.02	[0.27, 0.36]	0.19	0.04	[0.12, 0.25]	0.48	0.02	[0.45, 0.52]

**Table 2:** CNI parameter and 95% confidence intervals of the fear and neutral groups.

Group	C			N			I		
	*M*	*SD*	95%CI	*M*	*SD*	95%CI	*M*	*SD*	95%CI
Fear	0.30	0.02	[0.26, 0.35]	0.20	0.03	[0.13, 0.27]	0.58	0.02	[0.53, 0.62]
Neutral	0.27	0.02	[0.22, 0.32]	0.15	0.03	[0.08, 0.21]	0.48	0.02	[0.44, 0.52]

## Data Availability

The raw data supporting the conclusions of this article will be made available by the authors on request.
